# Human papillomavirus prevalence in South African women and men according to age and human immunodeficiency virus status

**DOI:** 10.1186/s12879-015-1181-8

**Published:** 2015-10-26

**Authors:** Zizipho Z A Mbulawa, David Coetzee, Anna-Lise Williamson

**Affiliations:** Institute of Infectious Disease and Molecular Medicine and Division of Medical Virology, University of Cape Town, Anzio Road, Observatory, 7925 Cape Town, South Africa; Center for HIV and STIs, National Institute for Communicable Disease, National Health Laboratory Service, Cape Town, South Africa; Centre for Infectious Disease Epidemiology and Research, School of Public Health and Family Medicine, University of Cape Town, Cape Town, South Africa; SAMRC Gynaecological Cancer Centre, University of Cape Town, Cape Town, South Africa; National Health Laboratory Service, Groote Schuur Hospital, Observatory, Cape Town, 7925 South Africa

**Keywords:** Human papillomavirus, Human immunodeficiency virus, Age

## Abstract

**Background:**

Both cervical cancer and human immunodeficiency virus (HIV) are major public health problems in Sub-Saharan Africa. The objectives of the study were to investigate human papillomavirus (HPV) prevalence according to age, HIV status and gender.

**Methods:**

Participants were 208 HIV-negative women, 278 HIV-positive women, 325 HIV-negative men and 161 HIV-positive men between the ages of 18–66 years. HPV types were determined in cervical and penile cells by Roche Linear Array HPV genotyping assay.

**Results:**

HPV prevalence was 36.7 % (76/207; 95 % confidence intervals (CI): 30.4–43.4 %) in HIV-negative women, with the highest prevalence of 61.0 % (25/41; 95 % CI: 45.7–74.4 %) in women aged 18–25 years. HPV prevalence was 74.0 % (205/277; 95 % CI: 68.5–78.8 %) in HIV-positive women, with the highest prevalence of 86.4 % (38/44; 95 % CI: 72.9–94.0 %) in women aged 18–25 years. HPV prevalence was found to decrease with increasing age in HIV-negative women (*P* = 0.0007), but not in HIV-positive women (*P* = 0.898). HPV prevalence was 50.8 % (159/313; 95 % CI: 45.3–56.3 %) in HIV-negative men, with the highest prevalence of 77.0 % (27/35; 95 % CI: 60.7–88.2 %) in men aged 18–25 years. HPV prevalence was 76.6 % (121/158; 95 % CI: 69.2–82.9 %) in HIV-positive men, with the highest prevalence of 87.5 % (7/8; 95 % CI: 50.8–99.9 %) in men 18–25 years of age. HPV prevalence was found to decrease with increasing age in HIV-negative men (*P* = 0.004), but not in HIV-positive men (*P* = 0.385). HIV-positive women had a significantly higher prevalence of one or more HPV type(s) in the bivalent (HPV-16/18: 20 % 55/277, 9 % 12/207; *P* <0.001), quadrivalent (HPV-6/11/16/18: 26 % 71/277, 12 % 24/207; *P* = 0.001) and nonavalent vaccine (HPV-6/11/16/18/31/33/52/56/58: 65 % 181/277, 24 % 50/207; *P* <0.001) compared to HIV-negative women. Similar observation were observed in men for bivalent (20 % 32/158, 10 % 30/313; *P* = 0.001), quadrivalent (35 % 56/158, 13 % 41/313; *P* <0.001) and nonavalent vaccine (75 % 119/158, 28 % 87/313; *P* <0.001).

**Conclusions:**

This study demonstrated high HPV prevalence among HIV-positive women and men in all age groups. The high prevalence of HPV types found in bivalent, quadrivalent and nonavalent vaccines in South African HIV-positive and HIV-negative women and men demonstrate that this population will greatly benefit from current HPV vaccines.

## Background

Worldwide 35.3 million people were living with human immunodeficiency virus (HIV) in 2012; 25 million in sub-Saharan Africa and 6.3 million in South Africa [1, 2]. HIV prevalence is higher in women than men. The peak in women is between 25 and 29 years while in men it is between 30 and 34 years of age [1–3]. In South Africa, women are more likely to have sexual partners who are 4 years older than themselves [4–7]. The median age at first sex in South African women ranges between 16 and 18 years while in men it ranges between 17 and 19 years of age [6, 8]. In a worldwide meta-analysis Africa was found to have the highest human papillomavirus (HPV) prevalence in women with normal cytology [9, 10]. Sub-Saharan Africa has the highest burden of HIV and cervical cancer and the epidemic of cervical cancer and HIV infection are major public health problems [2, 11].

Both HIV and HPV are sexually transmitted, with HPV being more infectious than HIV [12]. The prevalence of HIV and HPV is significantly higher among women and men with high-risk sexual behaviour [10]. HIV infection is significantly associated with a higher incidence of HPV, prevalence of HPV and persistence in women and men [13–15]. HIV-positive individuals are more likely to have multiple HPV infection and HPV viral load compared to their HIV-negative counterparts [16–19]. HPV-associated cancers occur more frequently in HIV-positive than in HIV-negative individuals [20, 21]. There is increasing evidence that HPV is associated with a two-three fold higher risk of HIV acquisition in both women and men [12, 22–30]. There are three HPV vaccines available, namely, bivalent vaccine (Cervarix®, GlaxoSmithKline) protecting against HPV 16/18, quadrivalent vaccine (Gardasil®, Merck), protecting against HPV 6/11/16/18 and nonavalent vaccine (Merck) protecting against HPV-6/11/16/18/31/33/52/56/58). These vaccines prevent infection and disease related to types they are targeting [31–36]. In March 2014, South Africa introduced vaccination with Cervarix® against HPV in schools, aiming to cover around 520 000 girls.

HPV prevalence decreases with increasing age in women [9, 37]. However, among older women inconsistent trends in HPV prevalence are observed, with some studies reporting a decrease or plateau in HPV prevalence while others report an increased HPV prevalence in older women [37]. HPV prevalence in women differs by country, region within the country and population group and this is likely to be due to the varying prevalence of high risk sexual behaviour worldwide [38]. HPV is acquired soon after sexual debut and the peak prevalence is observed in young women. The age distribution of HPV prevalence in African women is characterised by a U shape with high HPV prevalence in young and older ages [9, 38]. In sub-Saharan Africa genital HPV is reported to be very common in men [39, 40] and HPV prevalence does not decrease with age [41, 42]. The aim of this study is to demonstrate HPV prevalence, stratified by age and HIV status in women and men recruited from the same community.

## Methods

### Study population and specimen collection

The Research Ethics Committee of the University of Cape Town approved the study (reference: 258/2006) and written informed consent was obtained. Study participants were recruited from the Manyanani clinic, Empilisweni Centre, Gugulethu, Cape Town, South Africa between 2006 and 2009. Empilisweni is a wellness centre where HIV-related research is conducted. Individuals were recruited through clinics, bus stops and taxi ranks and eligible individuals were asked to come to Manyanani clinic. The study participants had to have had penetrative sexual intercourse with the partner of the opposite sex in the previous month to be enrolled in the study. Participation to the study was completely voluntary.

Cervical samples were collected by clinician using Digene cervical samplers. Clinicians collected penile samples by swabbing the penile shaft, glans, and foreskin with a dry Digene swab. Genital samples were stored in Digene transport medium at −80 °C until analysed. Study participants included 208 HIV-negative women, 278 HIV-positive women, 325 HIV-negative men and 161 HIV-positive men between the ages of 18 and 66 years. Cervical and penile samples were collected and stored as described by Mbulawa et al., [43].

### HPV genotyping

DNA was extracted by a MagNA Pure Compact (Roche) using the MagNA Pure Compact Nucleic Acid Isolation Kit (Roche). HPV genotyping was performed using the Roche Linear Array HPV genotyping test which identifies 37 different HPV genotypes. High-risk (HR) HPV types included HPV−16, −18, −31, −33, −35, −39, −45, −51, −52, −56, −58 and −59; probably or possible HR-HPV types included HPV−26, −53, −66, −67, −68, −70, −73 and −82; and low-risk (LR) HPV types HPV-6, −11, −40, 42, −54, −55, −61, −62, −64, −69, −71, −72, −81, −83, −84, −89 (HPV-CP6108) and –IS39 [44].

### Statistical analyses

Multiple HPV infection was defined as infection where two or more HPV types were detected. Cases with multiple infections were counted more than once when determining LR-HPV, HR-HPV, bivalent, quadrivalent or nonavalent prevalence. Statistical analysis was performed using chi-squared and chi-squared for trends (GraphPad prism 5). The 95 % confidence intervals of the proportion were calculated by modified Wald method (GraphPad Prism). Differences were considered to be statistically significant when *P*-values were <0.05.

## Results

### HPV prevalence in women according to age and HIV status

Demographic data are presented in Table 1. The median age was 37 years (18–66 years) for HIV-negative women; 32 years (18–65 years) for HIV-positive women; 38 years (19–67 years) for HIV-negative men and 36 years (22–64 years) for HIV-positive men. Table 2 presents the prevalence of any HPV, HR-HPV, probable HR-HPV, LR-HPV, multiple HPV infection and single HPV infection stratified by age and HIV status in women. Overall HPV prevalence in women was 58.1 % (281/484; 95 % CI: 53.6–62.4 %), with the highest prevalence of 74.1 % (63/85; 95 % CI: 63.9–82.3 %) observed in women 18–25 years of age and lowest prevalence of 50.0 % (67/134; 95 % CI: 41.7–58.4 %) in women 36–45 years of age. Age group 18–25 years were found to have significantly higher HPV prevalence compared to 26–35 years (74.1 % compared to 59.0 %, *P* = 0.017); to 36–45 years (74.1 % compared to 50.0 %, *P* = 0.0004) and to 46–66 years (74.1 % compared to 51.9 %, *P* = 0.0035). HPV prevalence was found to decrease with increasing age in women (*P* = 0.0032). The prevalence of multiple infections was 34.3 % (166/484) and of single infection was 23.8 % (115/484; Table 2).

**Table 1:** The demographic data of study participants

	HIV-negative women, *N* = 208	HIV-positive women, *N* = 278	HIV-negative men, *N* = 325	HIV-positive men, *N* = 161
Median age (range)	37 years (18–66 years)	32 years (18–65 years)	38 years (19–67 years)	36 years (22–64 years)
18–25 years	*N* = 41, median: 22 years	*N* = 44, median: 23 years	*N* = 35, median: 24 years	*N* = 8, median: 23 years
26–35 years	*N* = 59, median: 30 years	*N* = 130, median: 30 years	*N* = 99, median: 31 years	*N* = 64, median: 31 years
36–45 years	*N* = 64, median: 40 years	*N* = 71, median: 39 years	*N* = 105, median: 40 years	*N* = 67, median: 39 years
46–66 years	*N* = 44, median: 49 years	*N* = 33, median: 49 years	*N* = 86, median: 53 years	*N* = 22, median: 52 years
CD4 count				
≥350 ml^−1^	..	139 (50 %)	..	76 (47.2 %)
<350 ml^−1^	..	137 (49.3 %)	..	81 (50.3 %)
Missing	..	2 (0.7 %)	..	4 (2.5 %)
Age at first sex				
<16 years	28 (63.9 %)	49 (17.6 %)	87 (26.8 %)	40 (24.8 %)
16–18 years	123 (59.1 %)	171 (61.5 %)	163 (50.2 %)	94 (58.4 %)
>18 years	55 (26.4 %)	57 (20.5 %)	71 (21.8 %)	26 (16.2 %)
Missing	2 (1.0 %)	1 (0.4 %)	4 (1.2 %)	1 (0.6 %)
Smoking				
Never	133 (63.9 %)	182 (65.5 %)	48 (26.8 %)	31 (19.3 %)
Previously	13 (6.3 %)	28 (10.1 %)	45 (13.8 %)	26 (16.1 %)
Current	62 (29.8 %)	68 (24.5 %)	228 (70.2 %)	104 (64.6 %)
Missing	0 (0.0 %)	0 (0.0 %)	4 (1.2 %)	0 (0 %)
Lifetime number of sexual partners				
1–3	137 (65.9 %)	134 (48.2 %)	94 (28.9 %)	39 (24.2 %)
4–6	51 (24.5 %)	106 (38.1 %)	91 (28.0 %)	38 (23.6 %)
≥7	17 (8.2 %)	37 (13.3 %)	136 (41.8 %)	82 (50.9 %)
Missing	3 (1.4 %)	1 (0.4 %)	4 (1.2 %)	2 (1.2 %)
Cervical cytology				
Normal	157 (75.5 %)	167 (60.1 %)		
ASCUS	19 (9.1 %)	23 (8.3 %)		
LSIL	17 (8.2 %)	63 (22.7 %)		
HSIL	2 (1.0 %)	10 (3.6 %)		
Inadequate	13 (6.2 %)	14 (5.0 %)		
Missing	0 (0.0 %)	1 (0.4 %)		

*ASCUS* atypical squamous cell of undetermined significance, *LSIL* low grade squamous intraepithelial lesion, *HSIL* high grade squamous intraepithelial lesion

**Table 2:** Human papillomavirus (HPV) by age and human immunodeficiency (HIV) status in women

	All women	HIV-positive women	HIV-negative women	*P*-value^a^
	*N* = 484	*N* = 277	*N* = 207	
Any HPV				
All women	58.1 % (281/484)	74.0 % (205/277)	36.7 % (76/207)	**<0.0001**
*18–25 years*	*74.1 % (63/85)*	*86.4 % (38/44)*	*61.0 % (25/41)*	**0.008**
*26–35 years*	*59.0 % (111/188)*	*68.2 % (88/129)*	*39.0 % (23/59)*	**0.0002**
*36–45 years*	*50.0 % (67/134)*	*73.2 % (52/71)*	*23.8 % (15/63)*	**<0.0001**
*46–66 years*	*51.9 % (40/77)*	*81.8 % (27/33)*	*29.5 % (13/44)*	**<0.0001**
Multiple HPV infections				
All women	34.3 % (166/484)	49.8 % (138/277)	13.5 % (28/207)	**<0.0001**
*18–25 years*	*47.1 % (40/85)*	*61.4 % (27/44)*	*31.7 % (13/41)*	**0.007**
*26–35 years*	*34.6 % (65/188)*	*45.0 % (58/129)*	*11.9 % (7/59)*	**<0.0001**
*36–45 years*	*30.6 % (41/134)*	*49.3 % (35/71)*	*9.5 % (6/63)*	**<0.0001**
*46–66 years*	*26.0 % (20/77)*	*54.5 % (18/33)*	*4.5 % (2/44)*	**<0.0001**
Single HPV infection				
All women	23.8 % (115/484)	24.2 % (67/277)	23.2 % (48/207)	0.800
*18–25 years*	*27.1 % (23/85)*	*25.0 % (11/44)*	*29.3 % (12/41)*	0.664
*26–35 years*	*24.5 % (46/188)*	*23.3 % (30/129)*	*27.1 % (16/59)*	0.570
*36–45 years*	*19.4 % (26/134)*	*23.9 % (17/71)*	*14.3 % (9/63)*	0.161
*46–66 years*	*26.0 % (20/77)*	*27.3 % (9/33)*	*25.0 % (11/44)*	0.828
HR-HPV				
All women	39.5 % (191/484)	52.0 % (144/277)	22.7 % (47/207)	**<0.0001**
*18–25 years*	*54.1 % (46/85)*	*72.7 % (32/44)*	*34.1 % (14/41)*	**0.0004**
*26–35 years*	*37.2 % (70/188)*	*40.3 % (52/129)*	*30.5 % (18/59)*	0.199
36–45 years	*41.8 % (56/134)*	*64.8 % (46/71)*	*15.9 % (10/63)*	**<0.0001**
*46–66 years*	*24.7 % (19/77)*	*42.4 % (14/33)*	*11.4 % (5/44)*	**0.002**
Probable HR-HPV				
All women	21.1 % (102/484)	30.7 % (85/277)	8.2 % (17/207)	**<0.0001**
*18–25 years*	*25.9 % (22/85)*	*34.1 % (15/44)*	*17.1 % (7/41)*	0.080
*26–35 years*	*27.1 % (51/188)*	*37.2 % (48/129)*	*5.1 % (3/59)*	**<0.0001**
36–45 years	*15.7 % (21/134)*	*22.5 % (16/71)*	*7.9 % (5/63)*	**0.02**
*46–66 years*	*10.4 % (8/77)*	*18.2 % (6/33)*	*4.5 % (2/44)*	0.055
LR-HPV				
All women	34.5 % (167/484)	49.8 % (138/277)	14.0 % (29/207)	**<0.0001**
*18–25 years*	*44.7 % (38/85)*	*63.6 % (28/44)*	*24.4 % (10/41)*	**0.0003**
*26–35 years*	*36.7 % (69/188)*	*48.1 % (62/129)*	*11.9 % (7/59)*	**<0.0001**
36–45 years	*26.9 % (36/134)*	*43.7 % (31/71)*	*7.9 % (5/63)*	**<0.0001**
*46–66 years*	*31.2 % (24/77)*	*51.5 % (17/33)*	*15.9 % (7/44)*	**0.0006**

Number of infection will not always add up because participants with multiple infection were sometimes counted more than once. ^a^compares HIV-positive and HIV-negative women (chi-squared test)

HPV prevalence was 74.0 % (205/277; 95 % CI: 68.5–78.8 %) in HIV-positive women, with the highest prevalence of 86.4 % (38/44; 95 % CI: 72.9–94.0 %) in women 18–25 years of age and lowest prevalence of 68.2 % (88/129; 95 % CI: 60.0–75.6 %) in women 26–35 years of age. HIV-positive age group 18–25 years were found to have significantly higher HPV prevalence compared to 26–35 years (86.4 % compared to 68.2 %, *P* = 0.02); but not 36–45 years (86.4 % compared to 73.2 %, *P* = 0.1) and 46–66 years age group (86.4 % compared to 81.8 %, *P* = 0.59; Table 2). Among HIV-positive women, HPV prevalence did not decrease with increasing age (*P* = 0.898).

HPV prevalence was 36.7 % (76/207; 95 % CI: 30.4–43.5 %) in HIV-negative women, with the highest prevalence of 61.0 % (25/41; 95 % CI: 45.7–74.4 %) in women 18–25 years of age and lowest prevalence of 23.8 % (15/63; 95 % CI: 14.9–35.7 %) in women 36–45 years of age. HPV prevalence was found to be significantly higher in age group 18–25 years compared to 26–35 years (61.0 % compared to 39.0 %, *P* = 0.03); to 36–45 years (61.0 % compared to 23.8 %, *P* = 0.0002) and to 46–66 years (61.0 % compared to 29.5 %, *P* = 0.004; Table 2). HPV prevalence was found to decrease with increasing age in HIV-negative women (*P* = 0.0007). HIV-positive women were found to have significantly higher HPV prevalence compared to HIV-negative women in all age group (Table 2).

**Fig. 1:**
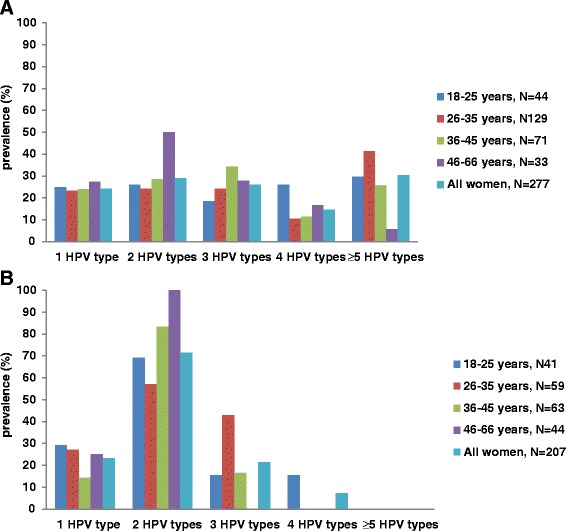
Distribution of multiple HPV genotype infections in HIV-positive (**a**) and HIV-negative (**b**) women

A higher proportion of detected HPV types in multiple infections than single infection were observed. The distribution of multiple infections (range: 2–14 HPV genotypes) is shown in Fig. 1a for HIV-positive women and Fig. 1b for HIV-negative women. Multiple HPV infections in HIV-negative women were more likely to be 2 HPV types while in HIV-positive women the distribution of multiple HPV types spread up over ≥5 HPV types.

### HPV prevalence in men according to age and HIV status

Table 3 presents prevalence of any HPV, HR-HPV, probable HR-HPV, LR-HPV, multiple HPV infection and single HPV infection stratified by age and HIV status in men. HPV prevalence in men was 59.4 % (280/471; 95 % CI: 55.0–63.8 %) with the highest prevalence of 79.1 % (34/43; 95 % CI: 64.6–88.8 %) in men 18–25 years of age and lowest prevalence of 47.6 % (50/105; 95 % CI: 38.3–57.1 %) in age. HPV prevalence was found to be significantly higher in age group 18–25 years compared to 26–35 years (79.1 % compared to 60.9 %, *P* = 0.03); to 36–45 years (79.1 % compared to 60.5 %, *P* = 0.02) and to 46–66 years (79.1 % compared to 47.9 %, *P* = 0.0005). HPV prevalence was found to decrease with increasing age in men (*P* = 0.0012). The prevalence of multiple infections was 41.0 % (193/471) and of single infection was 18.5 % (87/471) in men.

**Table 3:** Human papillomavirus (HPV) by age and human immunodeficiency (HIV) status in men

	All men	HIV-positive men	HIV-negative men	*P*-value^a^
	*N* = 471	*N* = 158	*N* = 313	
Any HPV				
All men	59.4 % (280/471)	76.6 % (121/158)	50.8 % (159/313)	**<0.0001**
*18–25 years*	*79.1 % (34/43)*	*87.5 % (7/8)*	*77.1 % (27/35)*	0.535
*26–35 years*	*60.9 % (95/156)*	*77.8 % (49/63)*	*49.5 % (46/93)*	**0.0004**
*36–45 years*	*60.5 % (101/167)*	*75.8 % (50/66)*	*50.5 % (51/101)*	**0.001**
*46–66 years*	*47.6 % (50/105)*	*71.4 % (15/21)*	*41.7 % (35/84)*	**0.015**
Multiple HPV infections				
All men	41.0 % (193/471)	62.7 % (99/158)	30.0 % (94/313)	**<0.0001**
*18–25 years*	*55.8 % (24/43)*	*87.5 % (7/8)*	*48.6 % (17/35)*	0.050
*26–35 years*	*44.2 % (69/156)*	*68.3 % (43/63)*	*28.0 % (26/93)*	**<0.0001**
*36–45 years*	*41.3 % (69/167)*	*59.1 % (39/66)*	*29.7 % (30/101)*	**0.0002**
*46–66 years*	*29.5 % (31/105)*	*47.6 % (10/21)*	*25.0 % (21/84)*	**0.041**
Single HPV infection				
All men	18.5 % (87/471)	13.9 % (22/158)	20.8 % (65/313)	0.071
*18–25 years*	*23.3 % (10/43)*	*0.0 % (0/8)*	*28.6 % (10/35)*	…
*26–35 years*	*35.9 % (56/156)*	*9.5 % (6/63)*	*21.5 % (20/93)*	0.050
*36–45 years*	*19.2 % (32/167)*	*16.7 % (11/66)*	*20.8 % (21/101)*	0.511
*46–66 years*	*18.1 % (19/105)*	*23.8 % (5/21)*	*16.7 % (14/84)*	0.453
HR-HPV				
All men	19.7 % (93/471)	54.4 % (86/158)	22.4 % (70/313)	**<0.0001**
*18–25 years*	*48.8 % (21/43)*	*75.0 % (6/8)*	*42.9 % (15/35)*	0.109
*26–35 years*	*39.1 % (61/156)*	*61.9 % (39/63)*	*23.7 % (22/93)*	**<0.0001**
36–45 years	*32.9 % (55/167)*	*50.0 % (33/66)*	*21.8 % (22/101)*	**0.0002**
*46–66 years*	*21.0 % (22/105)*	*28.6 % (6/21)*	*19.0 % (16/84)*	0.343
Probable HR-HPV				
All men	16.1 % (76/471)	54.4 % (86/158)	20.8 % (65/313)	**<0.0001**
*18–25 years*	*41.9 % (18/43)*	*62.5 % (5/8)*	*37.1 % (13/35)*	0.201
*26–35 years*	*28.2 % (44/156)*	*44.4 % (28/63)*	*17.2 % (16/93)*	**0.0002**
36–45 years	*29.9 % (50/167)*	*31.8 % (21/66)*	*28.7 % (29/101)*	0.671
*46–66 years*	*15.2 % (16/105)*	*42.9 % (9/21)*	*8.3 % (7/84)*	**<0.0001**
LR-HPV				
All men	29.5 % (139/471)	63.9 % (101/158)	33.2 % (104/313)	**<0.0001**
*18–25 years*	*53.5 % (23/43)*	*75.0 % (6/8)*	*48.6 % (17/35)*	0.187
*26–35 years*	*37.8 % (59/156)*	*66.7 % (42/63)*	*18.3 % (17/93)*	**<0.0001**
36–45 years	*47.9 % (80/167)*	*59.1 % (39/66)*	*40.6 % (41/101)*	**0.020**
*46–66 years*	*41.0 % (43/105)*	*66.7 % (14/21)*	*34.5 % (29/84)*	**0.008**

Number of infection will not always add up because participants with multiple infections were sometimes counted more than once. ^a^compares HIV-positive and HIV-negative men (chi-squared test)

HPV prevalence was 76.6 % (121/158; 95 % CI: 69.4–82.5 %) in HIV-positive men, with the highest prevalence of 87.5 % (7/8; 95 % CI: 50.8–99.9 %) in men 18–25 years of age and lowest prevalence of 71.4 % (15/21; 95 % CI: 49.8–86.4 %) in men 46–66 years of age. HPV prevalence was not found to be significantly higher in age group 18–25 years compared to 26–35 years (87.5 % compared to 77.8 %, *P* = 0.54); to 36–45 years (87.5 % compared to 75.8 %, *P* = 0.47) and to 46–66 years (87.5 % compared to 71.4 %, *P* = 0.39). HPV prevalence was not found to decrease with increasing age in HIV-positive men (*P* = 0.385); however, overall HPV prevalence and prevalence of multiple infections were high across all age groups (Table 3).

HPV prevalence was 50.8 % (159/313; 95 % CI: 45.3–56.3 %) in HIV-negative men, with the highest prevalence of 77.1 % (27/35; 95 % CI: 60.7–88.2 %) in men 18–25 years of age and lowest prevalence of 41.7 % (35/84; 95 % CI: 31.7–52.4 %) in men 46–66 years of age. HPV prevalence was found to be significantly higher in age group 18–25 years compared to 26–35 years (77.1 % compared to 49.5 %, *P* = 0.005); to 36–45 years (77.1 % compared to 50.5 %, *P* = 0.006) and to 46–66 years (77.1 % compared to 41.7 %, *P* = 0.0004). HPV prevalence was found to decrease with increasing age in HIV-negative men (*P* = 0.004). HIV-positive men were found to have significantly higher HPV prevalence compared to HIV-negative men in all age groups except for 18–25 years age group (Table 3).

**Fig. 2:**
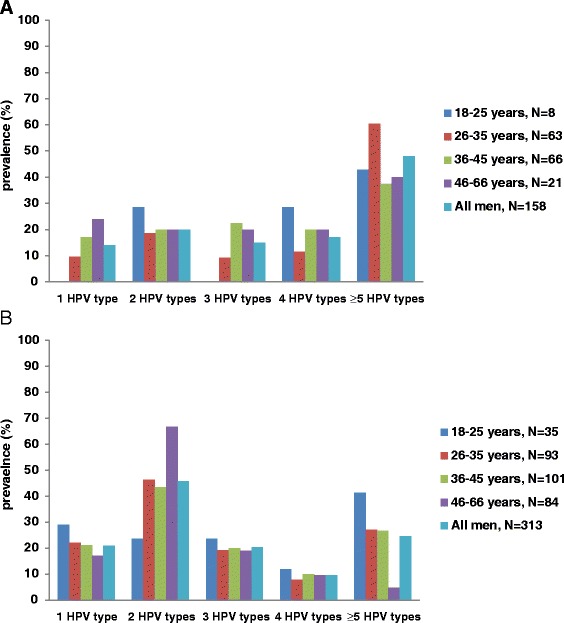
Distribution of multiple HPV genotype infections in HIV-positive (**a**) and HIV-negative (**b**) men

A higher proportion of detected HPV types were observed in multiple infections than single infection. The distribution of multiple infections (range: 2–13 HPV genotypes) is shown in Fig. 2a for HIV-positive men and Fig. 2b for HIV-negative men. Multiple HPV infections in HIV-negative men were more likely to be 2 HPV types while in HIV-positive men the distribution of multiple HPV types spread up to ≥5 HPV types.

### Prevalence of HPV types targeted by current HPV vaccines

**Fig. 3:**
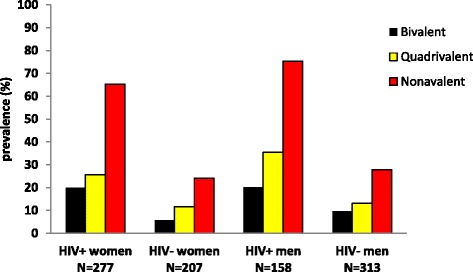
Prevalence of types targeted by bivalent, quadrivalent and nonavalent HPV vaccines in women and men according to HIV status

HIV-positive women had a significantly higher prevalence of one or more HPV types found in the bivalent vaccine (HPV-16/18: 20 % 55/277, 9 % 12/207; *P* <0.001), in the quadrivalent vaccine (HPV-6/11/16/18: 26 % 71/277, 12 % 24/207; *P* = 0.001) and in the nonavalent vaccine (HPV-6/11/16/18/31/33/52/56/58: 65 % 181/277, 24 % 50/207; *P* <0.001) compared to HIV-negative women (Fig. 3). HIV-positive men also had a significantly higher prevalence of one or more types found in the bivalent vaccine (20 % 32/158, 10 % 30/313; *P* = 0.001), in the quadrivalent vaccine (35 % 56/158, 13 % 41/313; *P* <0.001) and in the nonavalent vaccine (75 % 119/158, 28 % 87/313; *P* <0.001) compared to HIV-negative men (Fig. 3).

## Discussion

We previously reported the influence of HIV co-infection on HPV prevalence, acquisition and clearance in women and men [13, 43, 45, 46] and HPV concordance and transmission in sexual active couples [43, 46, 47]. The current report further demonstrates prevalence of any HPV type, LR-HPV, probable HR-HPV, LR-HPV, multiple HPV infections, single HPV infection and prevalence of HPV types targeted by bivalent, quadrivalent and nonavalent vaccines in women and men according to HIV status and age. HPV prevalence data specified by age and HIV status is important in understanding HPV trends especially in a country where HIV prevalence is very high. Women and men participated in this study were recruited as couples from the same community, a community with high prevalence of HIV (~30 % amongst pregnant women) and sexually transmitted infections [48, 49].

Higher prevalence of any HPV type, multiple infections, and types targeted by bivalent, quadrivalent and nonavalent vaccines in HIV-positives compared to HIV-negatives were observed. Generally, in women HPV prevalence decreased with age and this has been reported elsewhere [9, 38, 50–52]. However, when grouped according to HIV-status HPV prevalence decreased with age among HIV-negatives but not among HIV-positive women. In South African studies, HPV prevalence has also decreased with increasing age in HIV-negative women [53, 54] and in women not stratified by HIV status [55]. Notably in this study overall HPV prevalence and multiple infection prevalence remained high in HIV-positive women across all age groups while it decreased with age among HIV-negative women. McDonald et al., reported higher a HPV prevalence in South African among HIV-positive women compared to HIV-negative women in all age groups [54].

The higher prevalence of HPV in older HIV-positive women could be due to high rate of HPV reactivation as a result of suppressed immune system and susceptibility to new infections in HIV-positive women [17, 19]. According to Kjaer et al., HPV prevalence declines with age, even in highly sexually active women such as sex workers [52]. Some have suggested that older HIV-positive women are more likely to fail to clear the HPV infection they acquired at a young age or later, due to immune senescence [56, 57]. This is important as the persistence of genital HPV infection is associated with cervical disease progression [58]. A more intensive cervical screening program is required due to the high HPV prevalence and multiple infections across all age group among HIV-positive women.

HIV-prevalence was found to decrease with increasing age in men, when grouped according to HIV-status, this was also observed in HIV-negative men but not among HIV-positive men. In contrast, HPV prevalence in men has been reported not to vary with age [41, 42], however these reports were not reported according to HIV status. High HPV prevalence across age groups or slightly decrease as the age increases suggest high rate of persistent and acquired new HPV infections [42]. We previously reported high persistent and acquired new infection during follow-up among HIV-positive women and men in this cohort [13].

A high prevalence of infections with multiple HPV types was observed in HIV-positive women and men. HPV multiple infections are associated with a higher rate of persistent HPV infection than single infection [59]. Munagala et al., showed that persons with infections with multiple HPV types were more likely to have larger tumor and a poorer response to cancer treatment when compared with participants with single HPV infection [60]. HPV persistent infection is associated with the development of HPV related cancers in both women and men; while multiple HPV infection seem to complicate the response to treatment; and cancer prevention and treatment programs need to take this into consideration [59, 61]. The observations that single infection were more likely to be HR-HPV types than LR-HPV in women and not in men could be due to the fact that in women only the cervix was sampled. Jones et al., showed that LR-HPV were more likely to reside in the vagina than in the cervix [62]. In men several sites (penile shaft, glans and foreskin if uncircumcised) were sampled [43].

The prevalence of HPV types targeted by bivalence, quadrivalent and nonavalent HPV vaccines were found to be significantly higher among HIV-positives than HIV-negatives. Giuliano et al., reported a similar prevalence of one or more HPV types in quadrivalent (20 %) and nonavalent (28 %) vaccines in South African HIV-negative women [53].

The study was limited by the small sample size. Due to the small sample size the impact of HIV viral load and CD4 counts on HPV prevalence could not be stratified by age. All participants recruited for the study were volunteers and therefore not representative of the general population. Only participants who were sexually active were included and this was based on having had sexual intercourse in the previous month with a partner of the opposite sex and this was subject to recall bias. As this was a cross-sectional study a time sequence could not be interred.

## Conclusion

This study demonstrated high prevalence of HPV and multiple HPV infection among HIV-positive women compared to HIV-negative women across all ages. Older HIV-negative men had a higher prevalence of HPV compared to HIV-negative women of the same age group. The high prevalence of HPV types targeted by bivalent, quadrivalent and nonavalent vaccines in South African HIV-positive and HIV-negative women and men demonstrate that this population will greatly benefit from current HPV vaccines. Studies on HPV prevalence should be stratified by age and HIV status in both women and men.

## Abbreviations

CI: Confident interval
HIV: Human immunodeficiency virus
HPV: Human papillomavirus
HR: High-risk
LR: Low-risk
